# Supraspinal gene transfer by intrathecal adeno-associated virus serotype 5

**DOI:** 10.3389/fnana.2014.00066

**Published:** 2014-08-05

**Authors:** Daniel J. Schuster, Lalitha R. Belur, Maureen S. Riedl, Stephen A. Schnell, Kelly M. Podetz-Pedersen, Kelley F. Kitto, R. Scott McIvor, Lucy Vulchanova, Carolyn A. Fairbanks

**Affiliations:** ^1^Department of Neuroscience, University of MinnesotaMinneapolis, MN, USA; ^2^Department of Genetics, Cell Biology and Development, University of MinnesotaMinneapolis, MN, USA; ^3^Department of Pharmacology, University of MinnesotaMinneapolis, MN, USA; ^4^Department of Pharmaceutics, University of MinnesotaMinneapolis, MN, USA

**Keywords:** adeno-associated, AAV5, intrathecal, CNS, mannitol

## Abstract

We report the pattern of transgene expression across brain regions after intrathecal delivery of adeno-associated virus serotype 5 (AAV5). Labeling in hindbrain appeared to be primarily neuronal, and was detected in sensory nuclei of medulla, pontine nuclei, and all layers of cerebellar cortex. Expression in midbrain was minimal, and generally limited to isolated neurons and astrocytes in the cerebral peduncles. GFP immunoreactivity (-ir) in thalamus was most prominent in medial geniculate nucleus, and otherwise limited to posterior nuclei of the dorsal and lateral margins. Labeling was also observed in neurons and astrocytes of the hippocampal formation and amygdaloid complex. In the hippocampal formation, GFP-ir was found in neuronal cell bodies of the rostral ventral portion, but was largely restricted to fiber-like staining in the molecular layer of dentate gyrus and stratum lacunosum-moleculare of the rostral dorsal region. GFP-ir was seen in neurons and astroglia throughout caudal cortex, whereas in rostral regions of neocortex it was limited to isolated neurons and non-neuronal cells. Labeling was also present in olfactory bulb. These results demonstrate that intrathecal delivery of AAV5 vector leads to transgene expression in discrete CNS regions throughout the rostro-caudal extent of the neuraxis. A caudal-to-rostral gradient of decreasing GFP-ir was present in choroid plexus and Purkinje cells, suggesting that spread of virus through cerebrospinal fluid plays a role in the resulting transduction pattern. Other factors contributing to the observed expression pattern likely include variations in cell-surface receptors and inter-parenchymal space.

## Introduction

In recent years a growing body of evidence has indicated that adeno-associated virus (AAV) is a useful vector for delivery of therapeutic genes to the central nervous system (CNS). Direct injection of AAV vectors into a specific CNS nucleus can result in a high level of gene expression in that nucleus, as well as physically adjacent and functionally connected areas (Fu et al., 2002; Ciesielska et al., 2011; White et al., 2011). However, this method carries the drawback of tissue damage that is potentially both detrimental and irreparable. Therefore, alternative approaches for CNS delivery of AAV vectors, such as delivery to the cerebrospinal fluid (CSF), are necessary for translational development of AAV-mediated gene therapy.

Delivery to the CSF can be achieved in a variety of ways including injection into the cerebral ventricles, the posterior cistern, and the spinal intrathecal space. Acute injection of AAV vectors into the posterior cistern, without the use of any method to enhance distribution, has resulted in a relatively limited pattern of gene expression that appears analogous to that seen with ventricular injection (Fu et al., 2003). On the other hand, methods to improve vector distribution upon cisternal administration, such as pretreatment with mannitol delivered intravenously or removal of some CSF followed by a large injection volume, have shown the potential for an enhanced pattern of gene expression spanning several CNS regions (Fu et al., 2003; Iwamoto et al., 2009).

Surgical access to the posterior cistern has also been used for intrathecal catheter placement with the goal of delivery of AAV6 or AAV8 directed at the lumbar spinal level (Storek et al., 2008; Towne et al., 2009). Substantial transduction of lumbar dorsal root ganglion (DRG) neurons was achieved in both of these studies. Additionally, Towne et al. (2009) observed transduction in cervical DRG and several areas of the brain. An alternative method for delivery to the lumbar intrathecal space is direct lumbar puncture, which is minimally invasive, does not require anesthesia, presents negligible risk for tissue damage, and has long been used both experimentally and clinically.

We recently demonstrated transduction of DRG neurons following delivery of AAV5 or AAV8 by direct lumbar puncture with an intravenous mannitol pretreatment (Vulchanova et al., 2010). The goal of the current study was to determine the extent of expression in the CNS after lumbar puncture delivery of AAV5.

## Materials and methods

### AAV vector and packaging

AAV vector TRUF11, containing a CBA- (cytomegalovirus enhancer with a chicken β-actin promoter) regulated GFP sequence, has been previously described (Kaemmerer et al., 2000). Packaging using AAV5 serotype capsid was carried out at the University of Florida Vector Core Lab of the Gene Therapy Center (Gainesville, Florida) as previously described (Zolotukhin et al., 2002).

### Animals

Experimental subjects were 20–25 g adult male C57BL/6 mice (Harlan, Madison, WI). All experiments were reviewed and approved by the Institutional Animal Care and Use Committee (IACUC) of the University of Minnesota.

### Injections

Subjects were injected via the tail vein with 25% mannitol solution (200 μL) 20 min prior to intrathecal or intravenous injection of the viral construct. For intrathecal delivery, the AAV construct was delivered by direct lumbar puncture in awake mice by an experimenter (KFK) with extensive experience in this method of drug delivery (Hylden and Wilcox, 1980). In a minor modification to the protocol, the needle (30-gauge, 0.5-inch) was connected to a length of PE10 tubing, which was then connected to a second needle that was attached to a 50 μl Luer-hub Hamilton syringe. Ten microliters containing ~10^11^ vector genomes was injected intrathecally. The injection was administered by gently gripping the iliac crest of the rodent and inserting the needle (bevel side up) at about a 45° angle centered between the hipbones. A reflexive flick of the tail indicated puncture of the dura. For intravenous AAV5 injections, the same total dose of vector (~10^11^ vector genomes) was diluted 10-fold in sterile saline to a final volume of 100 μL. Following injection of AAV, the animals were returned to the vivarium where they remained for 6 weeks, until the time of transcardial perfusion, fixation, and extraction of fixed brain tissue for immunohistochemical analysis.

### Immunohistochemistry

All animals were sacrificed by perfusion fixation as previously described (Vulchanova et al., 1998). Briefly, animals were isoflurane anesthetized via nosecone inhalation and perfused with a solution of calcium-free tyrodes solution (in mM:NaCl 116, KCl 5.4, MgCl_2_·6H_2_0 1.6, MgSO_4_·7H_2_O 0.4, NaH_2_PO_4_ 1.4, glucose 5.6, and NaHCO_3_ 26) followed by fixative (4% paraformaldehyde and 0.2% picric acid in 0.1 M phosphate buffer, pH 6.9) followed by 10% sucrose in PBS. Brain was removed and incubated in 10% sucrose overnight at 4°C. Sections were cut at 14 μm thickness and thaw mounted onto gel-coated slides. Tissue sections were incubated for 1 h at room temperature in diluent (PBS containing 0.3% Triton, 1% BSA, 1% normal donkey serum) and then incubated overnight at 4°C in primary antisera diluted in the same solution. Primary antibodies used were: rabbit anti-GFP, 1:500 (Invitrogen; Eugene, OR), mouse anti-NeuN, 1:500 (Chemicon; Temecula, CA), mouse anti-GFAP, 1:400 (Sigma; St. Louis, MO), and mouse anti-calbindin, 1:1000 (Sigma; St. Louis, MO), rat anti-CD68, 1:100 (AbD Serotec, Oxford, UK). After rinsing with PBS, sections were incubated 1 h at room temperature with appropriate combinations of Cy2-, Cy3-, and Cy5- (1:300) conjugated secondary antisera (Jackson ImmunoResearch, West Grove, CA). Sections were rinsed again, and in some cases, were also incubated with DAPI nucleic acid stain for 3–5 min, 300 nM (Invitrogen; Eugene, OR). Following the final rinses, sections were cover-slipped using glycerol and PBS containing 0.1% p-phenylenediamine (Sigma).

### Microscopy

Anatomical analysis was based on the “The Mouse Brain In Stereotaxic Coordinates,” Second Edition (Paxinos and Franklin, 2001). Images for **Figures 3** and **10** were collected at a resolution of 1600 by 1200 pixels on an Olympus BX60 fluorescence microscope with a Spot Insight camera and Spot image acquisition software. Images in **Figures 4E,F**, **7** and **8B** were collected with an Olympus BX50FA fluorescence microscope equipped with a CCD camera. All other images were collected on an Olympus Fluoview 1000 confocal microscope with associated software at a resolution of 512 by 512 pixels. Images were processed for contrast, brightness, and color in Adobe Photoshop.

### Quantification

For quantification of number of fourth ventricle choroid plexus cells transduced, 3 sections per ventricle, spaced at least 100 μm apart were counted for each of 4 animals. The total number of choroid plexus cells in each section was determined by counting nuclei within each ventricle identified by DAPI stain. Choroid plexus cells immuno-positive for GFP were identified by overlaying images of DAPI fluorescence and GFP-ir, and counting nuclei that were surrounded by GFP-ir. Two independent observers performed counts yielding results that consistently varied by less than six percent per section. Results are graphed as percent of counted choroid plexus cells for a given ventricle that displayed GFP immunoreactivity (-ir).

For quantification of transduced Purkinje cells, at least 6 sections spaced at least 70 μm apart were counted for each of the 4 animals. Only Purkinje cell bodies that showed GFP-ir were counted, and were identified by co-labeling with calbindin-ir. Results are graphed as the percent of counted cells that were located in a given range of distances from Bregma.

### Statistics

For quantification of choroid plexus cells and Purkinje cells, data are represented as mean ± standard error of the mean. Data were analyzed using one-way analysis of variance (ANOVA) followed by a Bonferroni *post-hoc* test. Data analysis, and production of graphs, was done with GraphPad Prism 4.0 software.

## Results

Six weeks after intrathecal administration of AAV5, GFP expression was observed in discrete areas of the central nervous system (CNS), while intravenous delivery of an equivalent total dose of vector resulted in no detectable expression in the CNS. The figures illustrating localization of GFP-ir are presented in a caudal-to-rostral sequence and are referenced as necessary for the descriptions of anatomical regions, which may be represented on more than one figure. The relative abundance of GFP-positive cells in different structures is shown in Table 1. Throughout the CNS we observed GFP-positive neurons and astrocytes, as evidenced by colocalization with NeuN and GFAP, respectively. To address the immune status of the brains of AAV treated animals we performed labeling for the microglial marker CD68, and found no qualitative difference in CD68 labeling between naïve and AAV5 treated animals (data not shown).

**Table 1:** **Relative abundance of GFP-ir in brain after intrathecal AAV5-GFP delivery with an intravenous mannitol pretreatment (evaluated in 8 animals from two separate experiments; each structure was observed in at least 3 different sections)**.

**Brain area**	**Neuronal somata**	**Neuronal fibers**	**Non-neuronal cells**
Gracile/Cuneate n.	−	+++	−
Spinal Trigeminal n. and tract	+	++	−
NTS, Solitary tract, and AP	++	++	−
Vestibular/Cochlear n.	+	+	−
Facial n.	+	+	−
Cerebellum	++	++	−
4th Ventricle Choroid Plexus	−	−	++++
Pons	++	++	−
Inferior Colliculus	+	+	−
Superior Colliculus	+	+	−
Cerebral Peduncles	−	−	++
Medial Geniculate n.	++	++	−
LGN and Posterior Thalamic n.	+	++	−
Hypothalamus	+	+	−
3rd Ventricle Choroid Plexus	−	−	+++
Subiculum	+++	++	++
Entorhinal Ctx.	+++	++	++
Caudal visual & Retrosplenial Ctx.	++	++	++
Rostral visual & Retrosplenial Ctx.	+	+	+
Rostral−Ventral Hippocampal Form.	+++	++	+
Rostral−Dorsal Hippocampal Form.	−	+++	−
Amygdala	++	+	−
Lateral Ventricles Choroid Plexus	−	−	+++
Cingulate, Motor & Sensory Ctx.	+	+	+
Olfactory bulb	++	++	++

Definitions of relative abundance: −, no GFP labeling observed; +, isolated cells; ++, multiple labeled cells observed in close proximity to one another; +++, GFP-ir at least as prevalent as ++ throughout the structure, with some areas showing markedly increased GFP-ir; ++++ = a substantial fraction of cells throughout the entire structure showed GFP-ir. Abbreviations: n, nucleus; NTS, nucleus of the solitary tract; AP, area postrema; LGN, lateral geniculate nucleus; Ctx, cortex.

**Figure 1:**
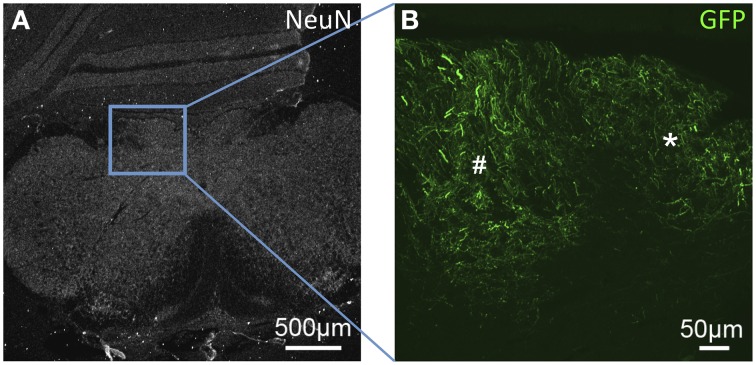
**GFP-ir fibers in somatosensory nuclei of the medulla. (A)** Low-magnification image showing the pattern of NeuN-ir in this tissue section (approximately −7.3 mm Bregma). **(B)** GFP-ir in the gracile (*) and cuneate (#) nuclei.

**Figure 2:**
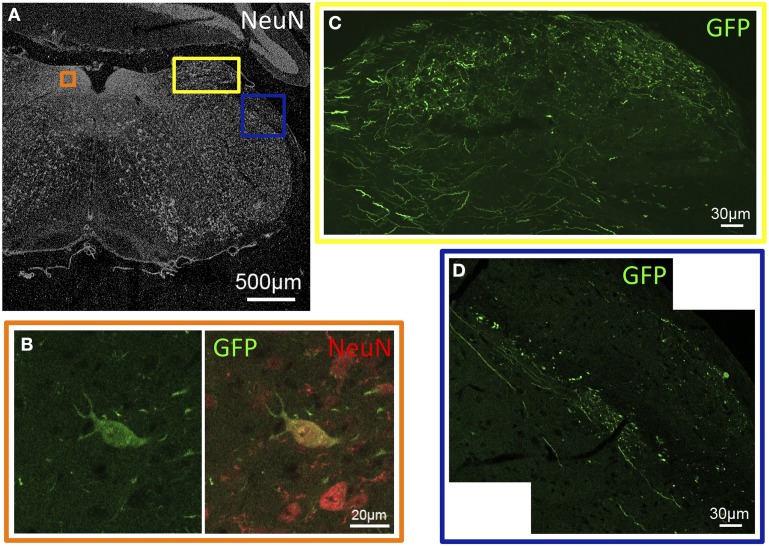
**GFP-ir in sensory nuclei of the medulla. (A)** Low-magnification image showing the pattern of NeuN-ir in this tissue section (approximately −6.8 mm Bregma). Colored inset boxes show the locations of the images in **(B–D)**. **(B)** A GFP-positive neuron in the nucleus of the solitary tract. **(C)** GFP-ir in the external cuneate nucleus. **(D)** GFP-ir in the spinal trigeminal tract (center diagonal) and lateral to that in the inferior cerebellar peduncle (right).

In the medulla, (as seen in Figures 1A, 2A) GFP-immunoreactivity (-ir) was found primarily in dorsal sensory, and cranial nerve nuclei. In the gracile, cuneate and external cuneate nuclei (Figures 1, 2C), as well as in the spinal trigeminal tract (Figure 2D), labeling was observed in neuronal fibers, but was absent from cell bodies. Fiber labeling in the gracile, cuneate, and external cuneate nuclei is likely to have originated from primary sensory neurons in dorsal root ganglia (DRG), as supported by our previously published observations of GFP-ir in nerve fibers within the dorsal columns of spinal cord (Vulchanova et al., 2010). In line with this observation, fibers in the spinal trigeminal tract are likely to have originated from neurons in the trigeminal ganglion. In vestibular nuclei and the solitary nuclear complex (Figure 2B), GFP-ir was found in neuronal cell bodies as well as fibers. GFP expression was also observed in cochlear and facial nuclei, and the area postrema (not shown).

**Figure 3:**
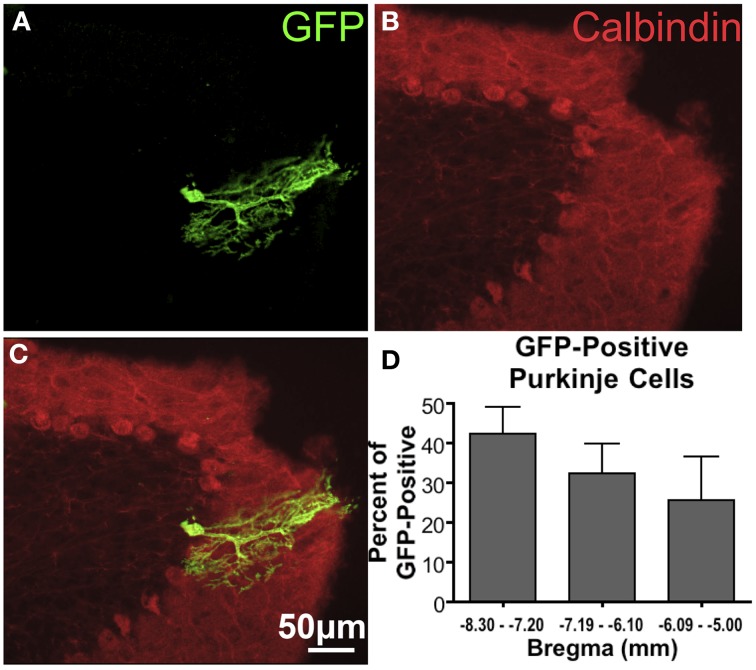
**GFP-ir in Purkinje cells of the cerebellum. (A)** GFP-ir, showing one labeled cell. **(B)** Calbindin-ir illustrating the difference in morphology between the molecular, Purkinje, and granule cell layers of the cerebellum. **(C)** Overlay of images in **(A)** and **(B)** illustrating that this GFP-positive neuron is a Purkinje cell. **(D)** A graph showing the fraction of GFP-positive Purkinje cells found at different distances from Bregma indicates a trend toward decreasing transduction of Purkinje cells in more rostral portions of cerebellum. Only GFP-positive Purkinje cells were counted. The graph represents counted cells for each range of distances from Bregma as percent of all GFP-positive Purkinje cells (78 total cells counted). There was no significant difference in the number of GFP-positive Purkinje cells found in each range of distances from Bregma (One-Way ANOVA, *P* > 0.05, *n* = 4).

In cerebellum, GFP-ir was observed in Purkinje cells (Figure 3), as well as cell bodies and fibers of the granular cell layer (not shown). Quantification of GFP-ir Purkinje cells co-labeled for calbindin showed a trend toward decreasing transduction of Purkinje cells in caudal-to-rostral fashion (Figure 3D). Although this trend did not reach significance, it supports the hypothesis that vector is distributed partially via rostral movement through CSF.

**Figure 4:**
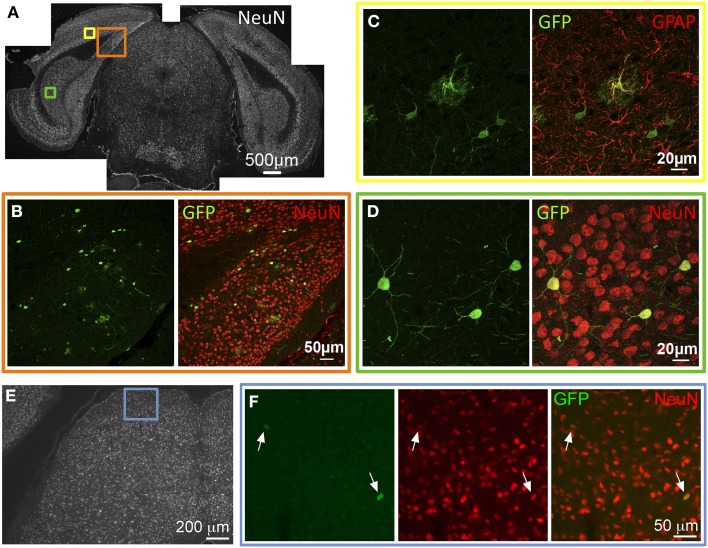
**GFP-ir in caudal cortex and midbrain. (A–D)** Co-localization of GFP with neuronal and astroglial markers in caudal cortex. **(A)** Low magnification image of NeuN-ir and colored inset boxes illustrate the locations of images in **(B–D)** (approximately −4.2 mm Bregma). **(B)** GFP-ir in cells across areas of primary visual cortex (v), retrosplenial cortex (rs), and presubiculum (prs). **(C)** Co-localization of GFP-ir with GFAP-ir illustrates that astrocytes are transduced, and can be distinguished by their morphology. **(D)** GFP-ir in neurons of the subiculum as evidenced by colocalization with NeuN-ir. **(E,F)** GFP-ir in isolated neurons in the superior colliculus. Arrows indicate GFP-positive neurons.

GFP expression was observed in several areas of the caudal cortices including primary and secondary visual cortex, retrosplenial cortex, presubiculum, parasubiculum, subiculum, and entorhinal cortex. In these locations, GFP-ir was seen in both neurons and astrocytes as evidenced by co-localization of GFP-ir with NeuN-ir or GFAP-ir respectively (Figures 4A–D). GFP-ir in midbrain, thalamus, and hypothalamus was predominantly limited to neurons, with the exception of astrocytes in the cerebral peduncles. In midbrain, GFP expression was minimal and observed primarily in isolated neurons within the intermediate gray matter of superior colliculus (Figures 4E,F).

**Figure 5:**
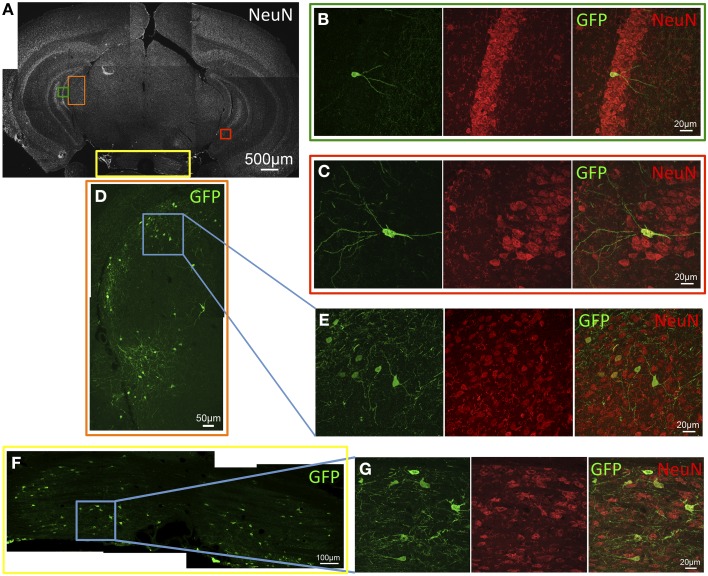
**GFP-ir in the caudal thalamus, hippocampal formation, and pons. (A)** Low magnification image of NeuN-ir and colored inset boxes illustrate the locations of images in B-G (approximately −3.0 mm Bregma). **(B–G)** Green shows GFP-ir, red shows NeuN-ir, and merged images illustrate co-localization in yellow. **(B)** A GFP-positive neuron in the granular layer of the dentate gyrus. **(C)** A GFP-positive neuron in the pyramidal layer of hippocampus area CA3. **(D)** GFP-ir in the medial geniculate nucleus (MGN) of the thalamus. **(E)** High magnification image of the area outlined by the blue box in D, illustrating that expression in the MGN is neuronal. **(F)** GFP expression in pontine nuclei. **(G)** High magnification image of the area outlined by the blue box in F, illustrating co-localization with NeuN-ir.

**Figure 6:**
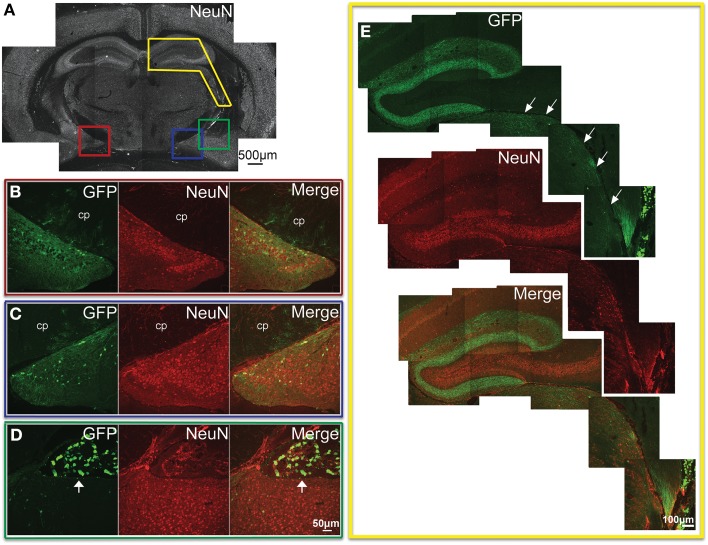
**GFP-ir in the hippocampal formation, amygdala, and thalamus. (A)** Low magnification image of NeuN-ir and colored inset boxes illustrate the locations of images in **(B–D)** (approximately −1.9 mm Bregma). **(B)** Substantial GFP-ir in neurons of the ventral dentate gyrus, ventral hippocampus, and superior to those, astrocytes in the cerebral peduncle (cp). **(C)** GFP expression in the amygdalohippocampal area, and superior to that, astrocytes in the cerebral peduncle (cp). **(D)** GFP-ir in the amygdaloid nuclear complex, and superior to that, in choroid plexus of the lateral ventricle (arrow). **(E)** GFP-ir in the dorsal hippocampal formation, and inferior and lateral to that (arrows), in thalamus including portions of the lateral geniculate, and posterior nuclei. Note that GFP-ir in the dorsal hippocampal formation appears restricted to fibers in the molecular layer of the dentate gyrus and the stratum lacunosum-moleculare of the hippocampus.

**Figure 7:**
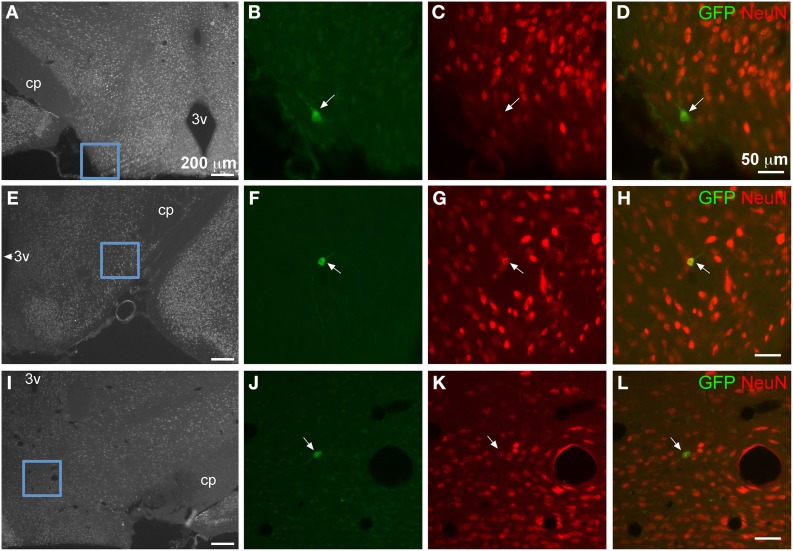
**GFP-ir in isolated neurons in hypothalamus**. Blue insets in **(A,E,I)** show the location of the corresponding higher magnification images relative to the third ventricle (3v) and the cerebral peduncle (cp). Green shows GFP-ir, red shows NeuN-ir, and merged images illustrate co-localization in yellow. Neurons within lateral hypothalamus are shown in **(B–D)** and **(F–H)**. **(J–L)** show a neuron in the supramammillary complex. All arrows indicate GFP-positive neurons.

In the most caudal portions of the hippocampus, (as seen in Figure 5A) GFP expression was found in granule cells of the dentate gyrus (Figure 5B), as well as pyramidal cells of the CA3 region (Figure 5C). The same tissue section shows GFP- ir in neurons of the medial geniculate nucleus (Figures 5D,E) of the thalamus, as well as in pontine nuclei (Figures 5F,G). The most rostral sections of hippocampus (as seen in Figure 6A) revealed many GFP-positive neuronal cell bodies only in the ventral portions (Figure 6B), whereas the dorsal portions contained primarily fiber-like staining in the molecular layer of the dentate gyrus and the stratum lacunosum-moleculare of the hippocampus proper (Figure 6E). Some GFP-expressing neurons were also observed in the amygdaloid nuclear complex (Figure 6C). A few neurons and some neuronal fibers were also observed in the pretectal, lateral geniculate, and posterior nuclei of thalamus (Figure 6E, dorsolateral surface of thalamus indicated by arrows). Labeling of non-neuronal cells, possibly astrocytes based on cell morphology similar to that shown in Figure 4C, in the cerebral peduncles is shown in Figures 6B,C (cp). In hypothalamus, GFP-ir was limited to isolated neurons (Figure 7).

**Figure 8:**
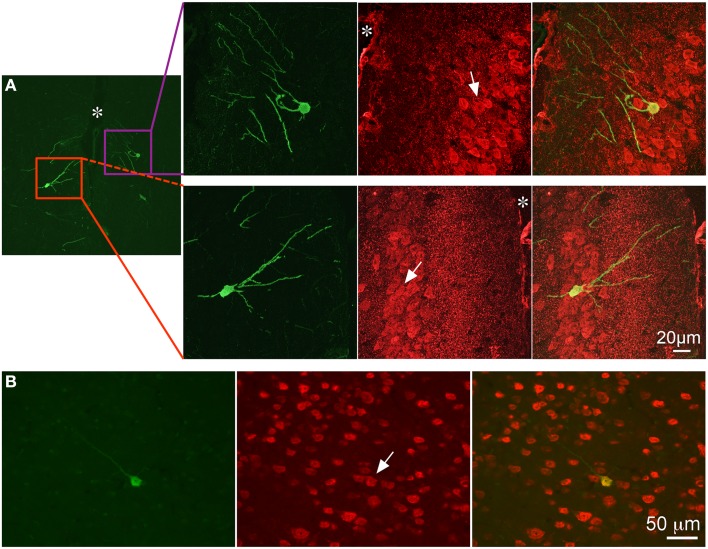
**GFP-ir in neocortex**. Colocalization of GFP-ir (green) and NeuN-ir (red) in cingulate cortex (**A**, ^*^ indicates the location of the median fissure) and somatosensory cortex **(B)**. All arrows indicate GFP-positive neurons.

**Figure 9:**
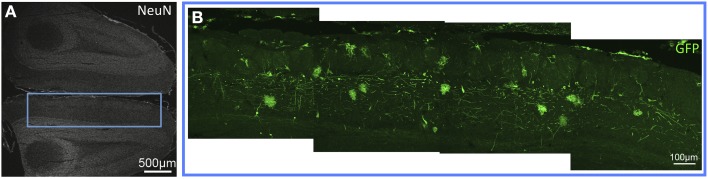
**GFP-ir in the olfactory bulb. (A)** Low magnification image showing NeuN-ir. **(B)** GFP-ir in neurons and astrocytes of the glomerular, external plexiform, and mitral cell layers of the medial olfactory bulb.

Evaluation of tissue sections in more rostral regions of neocortex revealed that GFP expression became less prevalent with labeling present only in isolated neurons and non-neuronal cells, which appeared to be astrocytes based on morphology. (Figure 8). GFP-ir was generally absent from striatum and minimal in surrounding neocortex. Sparse GFP-ir was seen in the islands of Calleja, the olfactory tubercles, and more rostrally in orbital cortex (not shown). In the olfactory bulbs expression of GFP was considerable, and was found in neurons and non-neuronal cells with astrocytic morphology in the medial portions of the glomerular, external plexiform, and mitral cell layers (Figure 9).

**Figure 10:**
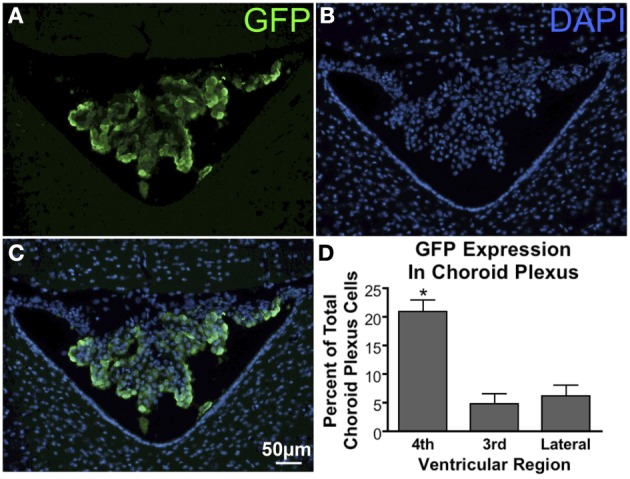
**GFP expression in the choroid plexus. (A)** GFP-ir in choroid plexus of the 4th ventricle. **(B)** DAPI staining in the same section showing cellular nuclei in the choroid plexus (center), cerebellum (top), and medulla (bottom left and right). **(C)** Overlay of images in **(A)** and **(B)** showing a substantial portion of the choroid plexus cells to be transduced. **(D)** Graph illustrating that transduction of choroid plexus cells is significantly higher in the 4th ventricle compared to the third and lateral ventricles. ^*^Indicates significance as determined by one-way ANOVA followed by Bonferroni *post-hoc* analysis, (*p* < 0.001, *n* = 4). Across all animals and ventricles 35,071 nuclei were counted, 3708 of which were identified as GFP-positive.

GFP-ir was evident in the choroid plexus (Figure 10 – 4th ventricle; Figure 6D, arrow – lateral ventricle), the structure that produces and secretes cerebrospinal fluid (CSF). Quantification of transduced choroid plexus cells throughout the ventricular system revealed that expression in the fourth ventricle was significantly higher than in the third or lateral ventricles (Figure 10D). This finding indicates that rostral movement through CSF from the injection site is an important factor in vector distribution.

## Discussion

In this report we have demonstrated AAV5-mediated gene transfer to the CNS using direct lumbar puncture intrathecal injection. Although GFP labeling was present throughout the rostro-caudal extent of the neuraxis, transgene expression was non-uniform and restricted to discrete brain regions. We have considered several factors that may influence the distribution of viral particles and the observed pattern of AAV5 transduction in the CNS.

We observed a caudal-to-rostral gradient of GFP expression within certain brain structures. For example, we found that a significantly larger portion of fourth ventricle choroid plexus cells express GFP relative to the third and lateral ventricles after AAV5. Considering the closer proximity of the fourth ventricle to the injection site, these results suggest that virus particles become less concentrated as they move through CSF rostral to the spinal column. Consistent with this observation, in cerebellum there appears to be a caudal to rostral trend from higher to lower number of GFP-expressing Purkinje cells.

The expression of vector genes or protein products in peripheral tissues suggests that intrathecally delivered viral vectors may also be distributed through the vascular system. We have consistently observed GFP expression in liver in our experiments (not shown), which is in line with previous reports of lumbar intrathecal AAV administration (Towne et al., 2009; Gray et al., 2013). These observations raise the possibility that some of the CNS expression observed may be the result of virus that has been distributed through the circulatory system. To address this hypothesis, we injected the same total dose of AAV5 that was given intrathecally, but delivered it intravenously after mannitol pretreatment. With this method we saw no detectable expression in the CNS (not shown). These results suggest that the vector does not regain access to the CNS after entering the peripheral circulation following intrathecal delivery at the doses we have used.

A remarkable finding that is not consistent with a simple caudal-to-rostral distribution of virus particles through the CSF is the relatively high (compared to other rostral forebrain regions) level of GFP expression in the medial portion of the olfactory bulb. Although the mechanism by which this level of expression is achieved is unknown, other groups have reported similar findings after injection of other AAV serotypes (AAV1 and AAV2) into either the posterior cistern or the lumbar intrathecal space (Fu et al., 2003; Watson et al., 2006; Iwamoto et al., 2009). Considering the position of the olfactory bulb in a small recess at the rostral end of the skull, it is possible that in this pocketed structure CSF flow may be restricted in a way that increases the retention of virus particles. In fact, it is known that CSF is able to drain along the olfactory nerve through the cribriform plate, and that this drainage constitutes a major pathway for CSF absorption in multiple species (Erlich et al., 1986; Williams and Blakemore, 1990; Kiwic et al., 1998; Mollanji et al., 2001). Significant CSF flow toward the olfactory nerve may cause virus particles following this current to get congested in the space near the olfactory bulb. An alternative explanation for the observed expression in olfactory bulb is that the cells may have migrated from the subventricular zone through the rostral migratory stream. We observed little to no GFP-ir in the subventricular zone (not shown); however, as tissue was examined 6 weeks after vector injection, we cannot rule out the possibility that GFP-positive cells in olfactory bulb may have been transduced in the subventricular zone prior to migration through the rostral migratory stream.

Another brain region with a noteworthy expression pattern that was non-uniform within the structure was the hippocampal formation. GFP-positive cell bodies (primarily neuronal) were found in the hippocampal formation, and were most prevalent in the rostral-ventral region. Although GFP-ir was also present in the rostral-dorsal hippocampal formation, labeling was primarily neuropil-like, and rarely found in neuronal cell bodies. This suggests that the fiber staining originates from neuronal cell bodies in another location, most likely the entorhinal cortex, where GFP-positive neurons were observed. It is unclear why neurons in ventral hippocampus were transduced much more efficiently that in dorsal hippocampus. One possible explanation is variation in the level of expression of cell surface receptors for virus particles. For AAV5, there are two known receptors: α2,3 N-linked sialic acid and platelet-derived growth factor receptor (PDGFR) types alpha and beta (Walters et al., 2001; Di Pasquale et al., 2003). There is currently no literature directly describing the pattern of expression of α2,3 N-linked sialic acid in the mouse brain. We have previously used lectin-histochemistry to investigate α2,3 N-linked sialic acid in DRG and observed diffuse staining with no apparent relationship between labeling intensity and expression of vector-delivered GFP (unpublished observations). For both PDGFR alpha and beta, high expression levels have been reported in CNS regions where AAV5 transduction was limited or absent in our experiments, suggesting that PDGFR is not the limiting factor for transduction by AAV5 in the CNS (Fruttiger et al., 1999; Ishii et al., 2006). Though we cannot make a direct link between AAV5 receptor expression and our observed transduction pattern, the dorsal and ventral hippocampus have been shown to differ both in function and molecular expression (Fanselow and Dong, 2010). Additionally, we have previously shown in dorsal root ganglia that AAV5 differentially targets subpopulations of DRG neurons that are defined by differences in molecular expression and function (Vulchanova et al., 2010). Our observations in the hippocampal formation may also reflect AAV vector tropism for specific cellular subpopulations.

One anatomical feature of the CNS that could explain differences in expression across brain regions is parenchymal permeability due to differences in the structure of the pia mater. Although it has been reported that the organization of the pia mater varies substantially (Morse and Low, 1972), this variability has not been examined systematically. Moreover, there is evidence for the presence of fenestrations within the pia mater, which, at least at the level of spinal cord, appear to be differentially distributed (Reina et al., 2004). Interestingly, the lack of fenestrations at thoracic levels correlates with the reduced transduction frequency at thoracic levels compared to lumbar and cervical levels (Towne et al., 2009; Vulchanova et al., 2010), supporting the idea that differential membrane permeability may influence the distribution of viral vector delivered intrathecally.

In summary, the pattern of transduction in the CNS following intrathecal administration of an AAV vector with an intravenous mannitol pretreatment appears to be the result of several factors discussed above, including: rostral movement through CSF, overall CSF flow, membrane permeability, and expression of cell surface receptors for virus particles. The current study has revealed that under these specific spinal drug delivery conditions, discrete brain areas appear to be transduced by AAV5.

## Author contributions

Daniel J. Schuster participated in perfusions and dissections, performed immunohistochemistry, imaging, cell quantification, and some of the intravenous injections. He analyzed the data, prepared the figures, participated in interpretation of the results, and drafted and edited the manuscript. Lalitha R. Belur initiated the studies and contributed to the experimental design and edited the manuscript. Maureen S. Riedl performed all perfusions and dissections and participated in histochemical analyses and interpretation of results. Stephen A. Schnell contributed to data analysis and figure preparation. Kelly M. Podetz-Pedersen performed some of the IV injections. Kelley F. Kitto conducted all intrathecal injections of vector and is responsible for the quality assurance of this key technique. R. Scott McIvor initiated the study with CF as collaborators and edited the manuscript. Lucy Vulchanova participated in all perfusions and dissections, as well as contributed to the experimental design, histochemical analyses, interpretation of results, generation of figures, and editing of the manuscript. Carolyn A. Fairbanks organized the team, contributed to the experimental design, edited the manuscript, and supported the studies.

### Conflict of interest statement

The authors declare that the research was conducted in the absence of any commercial or financial relationships that could be construed as a potential conflict of interest.
